# 3-D breast nodule detection on automated breast ultrasound using faster region-based convolutional neural networks and U-Net

**DOI:** 10.1038/s41598-023-49794-8

**Published:** 2023-12-18

**Authors:** Kangrok Oh, Si Eun Lee, Eun-Kyung Kim

**Affiliations:** 1 Research Institute of Radiological Science and Center for Clinical Imaging Data Science, Yonsei University College of Medicine, Seoul, 03722 Republic of Korea; 2 Department of Radiology, Yongin Severance Hospital, Yonsei University College of Medicine, 363, Dongbaekjukjeon-daero, Giheung-gu, Yongin, Gyeonggi-do 16995 Republic of Korea

**Keywords:** Breast cancer, Cancer

## Abstract

Mammography is currently the most commonly used modality for breast cancer screening. However, its sensitivity is relatively low in women with dense breasts. Dense breast tissues show a relatively high rate of interval cancers and are at high risk for developing breast cancer. As a supplemental screening tool, ultrasonography is a widely adopted imaging modality to standard mammography, especially for dense breasts. Lately, automated breast ultrasound imaging has gained attention due to its advantages over hand-held ultrasound imaging. However, automated breast ultrasound imaging requires considerable time and effort for reading because of the lengthy data. Hence, developing a computer-aided nodule detection system for automated breast ultrasound is invaluable and impactful practically. This study proposes a three-dimensional breast nodule detection system based on a simple two-dimensional deep-learning model exploiting automated breast ultrasound. Additionally, we provide several postprocessing steps to reduce false positives. In our experiments using the in-house automated breast ultrasound datasets, a sensitivity of $93.65\%$ with 8.6 false positives is achieved on unseen test data at best.

## Introduction

Breast ultrasound is a widely employed imaging modality for cancer detection and diagnosis. Specifically, breast ultrasound has been used as an attractive supplementary imaging modality to mammography for dense breasts^1^. Conventional two-dimensional (2-D) hand-held ultrasound (HHUS) imaging has limitations such as high operator dependence, long examination time, and poor reproducibility^2^. Recently automated breast ultrasound (ABUS) imaging has been an emerging modality resolving the limitations of HHUS^3–11^. ABUS imaging system produces volumetric images which enable new diagnostic information, i.e., coronal plane reconstruction^12^. However, ABUS imaging requires a long interpretation time due to the vast amount of images for the diagnosis. Hence, developing computer-aided detection (CADe) systems for ABUS is critical and valuable in the field.

Breast nodule detection has been investigated thoroughly for HHUS images, where the applied techniques are effective for those using ABUS images. Yap et al.^13^ proposed to adopt patch-based LeNet^14^, U-Net^15^, and a transfer learning approach based on pre-trained fully connected networks (FCN-AlexNet^16,17^) for breast lesion detection. Cao et al.^18^ presented an experimental study comparing the fast region-based convolutional neural networks (Fast R-CNN)^19^, Faster R-CNN^20^, you only look once (YOLO)^21^, YOLO version3 (YOLOv3)^22^, and single shot multibox detector (SSD)^23^ for breast lesion detection. Similarly, Yap et al.^24^ proposed a transfer learning approach based on the Faster R-CNN, and Li et al.^25^ proposed a deep learning model based on unsupervised region proposal and the ResNet-50^26^ architecture for breast lesion detection. These state-of-the-art studies regarding breast nodule detection using HHUS images adopted advanced deep-learning models to guarantee a certain level of detection performance and generalization capability.

Those pioneering studies^6,27^ regarding breast nodule detection for ABUS images utilized a combination of image pre-processing, feature extraction, and classification techniques such as multi-scale blob detection^6^, and watershed transform^28^. These studies tend to show low generalization capability, while various deep-learning models were adopted and proposed in recent studies. For 2-D breast lesion detection for ABUS images, Zhang et al.^29^ introduced a Bayesian YOLO version4 (YOLOv4) architecture by applying the uncertainty scheme into the YOLOv4^30^ model. However, this study is limited to 2-D lesion detection using ABUS images. To detect three-dimensional (3-D) tumors in ABUS images, Moon et al.^31^ and Chiang et al.^32^ proposed systems based on 3-D convolutional neural networks (CNN). Lei et al.^33,34^ presented a 3-D patch-based Mask scoring R-CNN for 3-D breast tumor segmentation, while the study focused on solving the segmentation problem. Because the aforementioned 3-D breast nodule detection systems were constructed using 3-D patch-based CNN architectures, they inevitably operate in a sliding window scheme at the testing phase to locate suspicious breast lesions. Locating 3-D breast nodules using 2-D was investigated in several studies. Zhang et al.^35^ adopted YOLO version5 (YOLOv5)^36^ as a detector and 3-D ResNet as a classifier to reduce false positives (FPs). A convolutional BiLSTM network was proposed by Malekmohammadi et al.^37^, where the approximate location of 3-D breast lesions was provided as the format of a heat map. Zhou et al.^38^ investigated a fusion of multi-view 2-D breast tumor detection outcomes by modifying Faster R-CNN architecture for 3-D breast tumor detection.

In this study, we aim to detect 3-D breast nodules using a simple 2-D deep learning methodology. To accomplish this goal, we employ the Faster R-CNN model due to the advantages of sharing convolutional features for region proposal and object detection and avoiding excessive search by a sliding window strategy. Subsequently, we propose a couple of additional postprocessing steps for removing false positives (FPs) to improve the detection performance. In addition, transverse, coronal, and sagittal plane images are utilized as input images because it is an advantage of ABUS volumetric imaging.

## Methods

**Figure 1:**
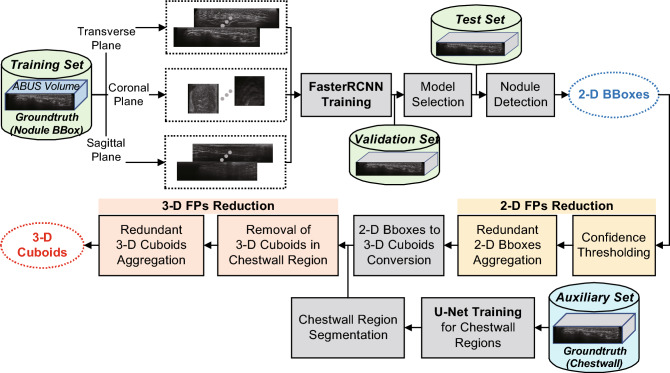
An overview of the proposed 3-D breast nodule detection system.

In this study, we propose an ABUS-based 3-D breast nodule detection system. The faster region-based convolutional neural networks (Faster R-CNN) model^20^, which has been frequently employed for breast nodule detection on HHUS and ABUS images^18,38^, is adopted to locate suspicious breast nodules from 2-D images in transverse, coronal, and sagittal planes. The 2-D FPs and redundant bounding boxes are eliminated based on a thresholding operation on output confidence values and clustering technique. Reduced 2-D bounding boxes extracted from 2-D image slices are then converted to 3-D cuboids based on a set of computations using row, column, and slice coordinates. Subsequently, redundant and FP 3-D cuboids are excluded based on U-Net segmentation model^15^ and coordinates processing. Consequently, breast nodules in the form of 3-D cuboids are detected via the aforementioned 2-D deep learning methodology and postprocessing. Figure 1 shows an overview of the proposed 3-D breast nodule detection system.

### Automated breast ultrasound data acquisition and preprocessing

We acquired ABUS images from Yongin Severance Hospital, Yonsei University College of Medicine, South Korea, to evaluate breast nodule detection performance. The Yongin Severance Hospital Institutional Review Board approved this study (IRB Number: 9-2021-0107). All research was performed under relevant guidelines and regulations. Informed consent was waived by the Institutional Review Board because of the retrospective nature of the study, and the analysis used anonymous clinical data. The capturing device is Invenia ABUS 2.0 from GE Healthcare for entire ABUS images. The captured ABUS scans have the form of stacks of 2-D images in the transverse plane. The 2-D image in the transverse plane has the size of $546 \times 843$, where the number of transverse slices is around 350. Pixel spacing values in the longitudinal, lateral, and vertical axes are 0.073, 0.2, and 0.4757 millimeters. Resampling is performed to reformat the ABUS images as isotropic scans with 0.2 mm voxel spacing. Consequently, the reformatted ABUS scans have the size of $198 \times 842 \times 830$ pixels. Loading DICOM files and reformatting them to a stack of gray-scale images with isotropic voxel spacing was performed using MATLAB R2022a^39^. Specifically, the ‘dicomread’ and ‘interp3’ functions are the main components for processing. For the PyTorch implementation of the Faster R-CNN model for gray-scale images, converting the gray-scale images to RGB images by repeatably concatenating the gray-scale images according to Yap et al.^24^, and rescaling the intensity values within $\left[ 0,1\right]$ was performed.

The in-house ABUS dataset A acquired from April 2020 to January 2021 for training and testing consists of 308 scans/passes from 103 female patients. It contains 240 scans with benign-appearing nodules (Breast Imaging Reporting and Data System (BI-RADS)^40^ categories 2 and 3) from 74 patients and 68 scans with suspicious nodules (BI-RADS categories 4 and 5) from 29 patients. These 29 patients may have nodules with benign features concurrently. The range of patients’ ages is between 23 and 87 years, and the average age is 52.9. Each breast has three scans at maximum to capture the entire breast region and scans with at least one breast nodule (BI-RADS 2, 3, 4, and 5) were included in the in-house ABUS dataset A. Because we limit our study to detect nodules within BI-RADS categories 3, 4, and 5 (55, 90, and 19 nodules, respectively) due to their clinical impact, breast nodules with BI-RADS category 2 is not the target of detection. Radiologist A (with four years of experience) participated in annotating breast nodules in the in-house ABUS datasets A. We used the in-house ABUS dataset A for model development and cross-validation tests.

Additionally, the in-house ABUS dataset B has been acquired from February to June 2021 as a separate test dataset, which is an alternative to an external validation dataset under our limited circumstances. The in-house ABUS dataset B consists of 168 scans from 28 female patients. It contains 126 scans with benign-appearing nodules (BI-RADS categories 2 and 3) from 21 female patients and 42 scans with suspicious nodules (BI-RADS categories 4 and 5) from 7 female patients. The six scans covering the entire breast regions per patient were included in the in-house ABUS dataset B. Hence, it may contain scans capturing the breast region without nodules, unlike the in-house ABUS dataset A. The in-house ABUS dataset B includes 23, 34, and 6 nodules in BI-RADS categories 3, 4, and 5, respectively. Radiologist B (with 27 years of experience) participated in annotating breast nodules in the in-house ABUS dataset B to secure diversity and prevent possible bias. Moreover, an auxiliary ABUS dataset was acquired in February 2023 for chest wall region segmentation to remove FPs using the U-Net model. The auxiliary ABUS dataset consists of 480 images in the transverse plane from 8 subjects, where 10 images were randomly chosen from each scan. Radiologist A participated in marking the boundary of the chest wall area.

### Faster R-CNN training

**Figure 2:**
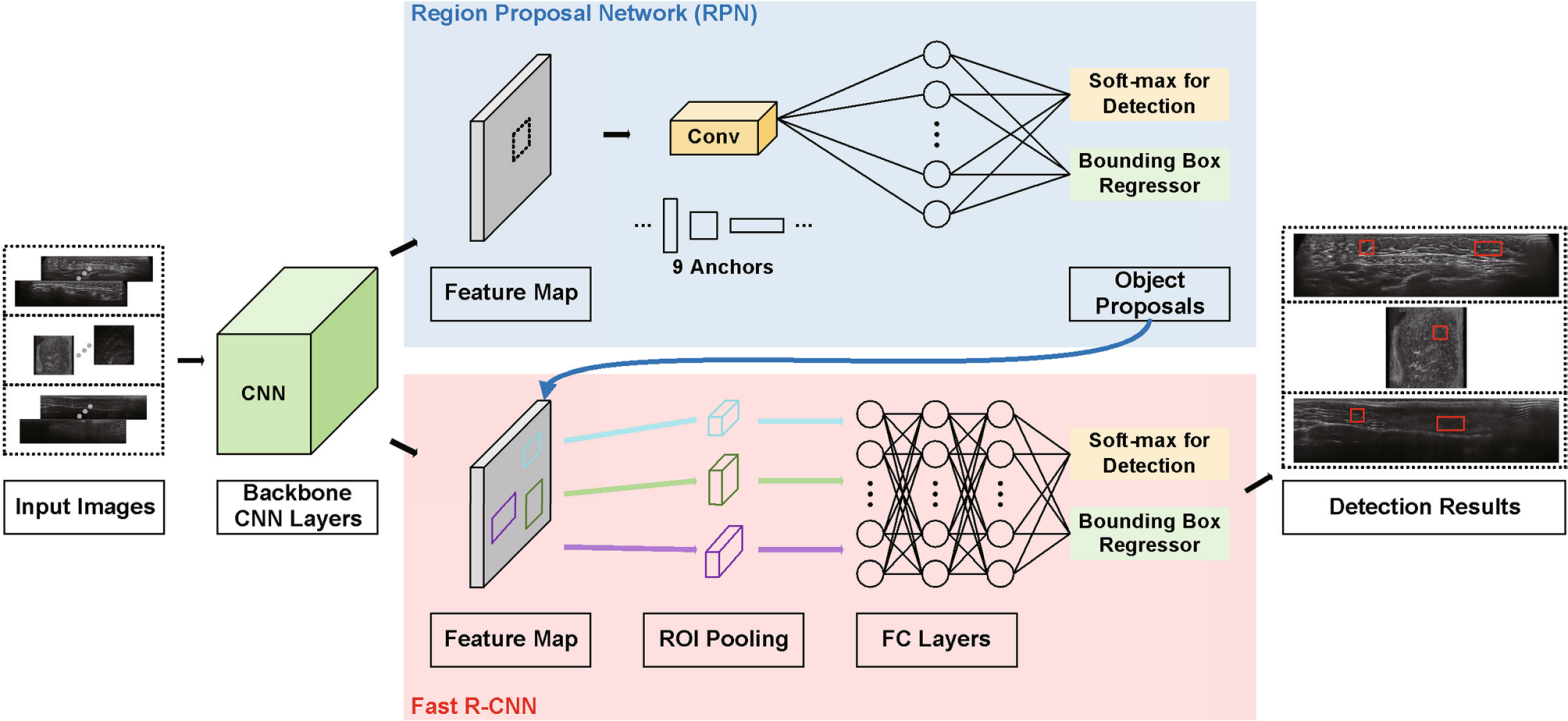
A simplified diagram of the Faster R-CNN.

In our application scenario, we utilize 2-D images in the transverse, coronal, and sagittal planes separately to build the breast nodule detector. For this, we adopt the Faster R-CNN due to its impressive speed and accuracy performance demonstrated in the object detection tasks in medicine^29,41–44^. The Faster R-CNN attempts to solve the computational bottleneck of the object detection networks based on region proposal algorithms^19,45^. Specifically, a region proposal network (RPN) and an object detection network are trained end-to-end via a simple alternating optimization by sharing convolutional features. This aspect enables nearly cost-free region proposals. Figure 2 illustrates a simplified flow diagram of the Faster R-CNN applied in breast nodule detection. According to^46^, region proposal-based object detectors such as the Faster R-CNN tend to be more efficient in speed than those based on the sliding window and CNNs. In order to reduce the burden of retaining a large number of training examples while increasing the generalization capability, we utilized a ResNet-50 model^45^ pre-trained using the Microsoft COCO dataset^47^ as the backbone CNN architecture. With such setting, the Faster R-CNN can be effectively trained using relatively small data. The standard nine anchor boxes are utilized for the RPN. For optimization, we adopted the stochastic gradient descent^48^ with a learning rate of 0.001 and momentum of 0.9. The learning rate was set to diminish by a factor of 0.1 for every three epochs. We set the number of epochs to 25 and the weight decay factor to 0.0005.

Since the in-house ABUS dataset consists of volumetric image data, the Faster R-CNN detector produces multiple 2-D detection outcomes for each slice. Hence, locating a 3-D breast nodule essentially requires additional processing, which are summarized as (1) removing FP bounding boxes in 2-D slice images and aggregating redundant 2-D bounding boxes, (2) establishing links between 2-D bounding boxes from sequential slices and generating 3-D cuboids, and (3) removing FP cuboids in chest wall regions and aggregating redundant 3-D cuboids.

**Figure 3:**
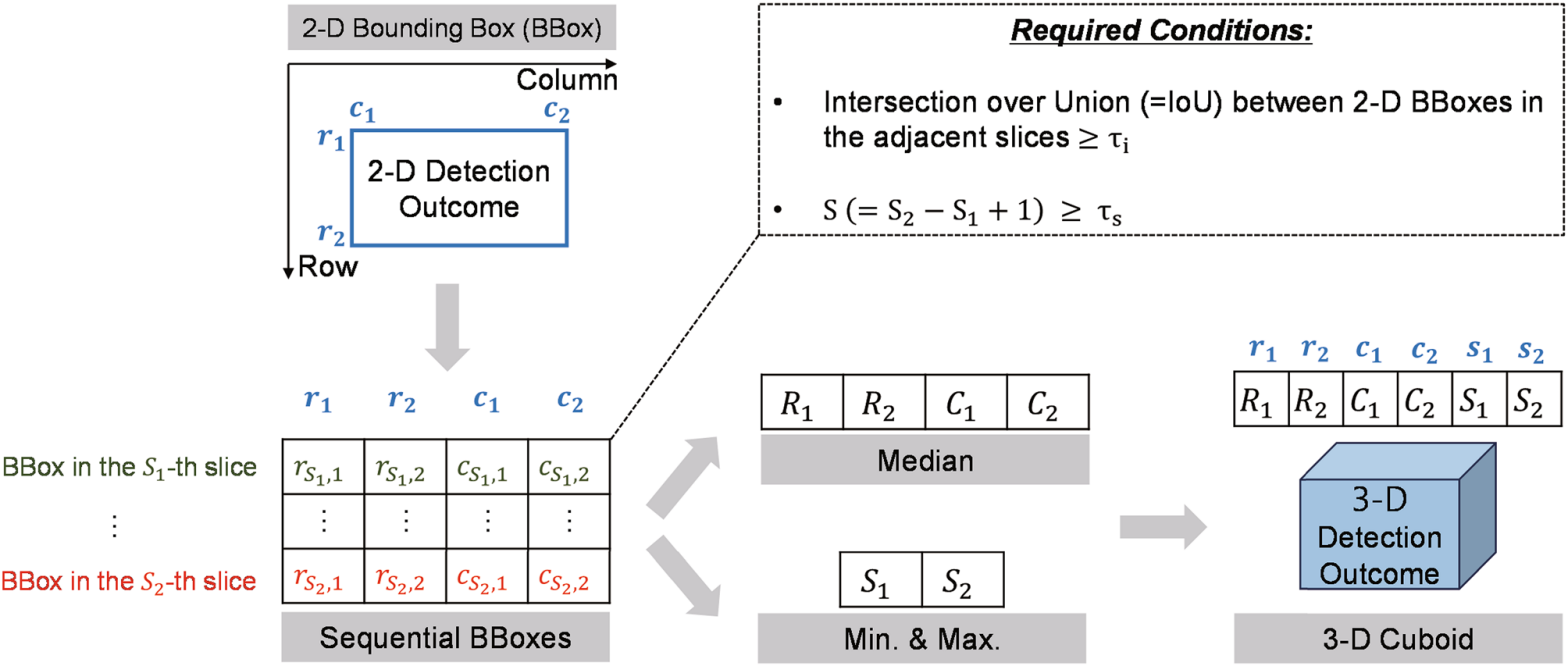
A flow diagram for 2-D bounding boxes to 3-D cuboids conversion process.

### 2-D false positives reduction

Because the 2-D detection outcomes include bounding boxes with significantly small confidence values, a threshold $\tau _{c}$ is defined to remove weak detection outcomes considered FPs. Subsequently, we utilize hierarchical clustering^49^ to aggregate redundant 2-D bounding boxes detected in a single slice image. As a similarity metric between two bounding boxes, the intersection over union (IoU) criterion^50^ is utilized in performing the hierarchical clustering technique. As a cut-off value, we set 0.3 to decide the bounding boxes are associated. Consequently, the representative bounding box of a cluster is defined by calculating the median coordinates of associated bounding boxes. The median operation is performed per each corner of the associated bounding boxes.

### 2-D bounding boxes to 3-D cuboids conversion

The 3-D breast nodule cuboid is located by aggregating 2-D bounding box information extracted from 2-D image slices. Considering every pair of adjacent 2-D slice images, sequential 2-D bounding boxes with an IoU greater than or equal to a threshold $\tau _i$ are regarded as connected. If the number of slices with connected bounding boxes is higher than or identical to a threshold $\tau _s$, the sequential 2-D bounding boxes are converted to a 3-D cuboid. Otherwise, they are assumed small and excluded for further processing. Because the connected 2-D bounding boxes are located irregularly throughout the sequential slice images, 3-D cuboid information is constructed by obtaining 2-D median row and column coordinates of the four corners of connected 2-D bounding boxes and the minimum and maximum slice numbers. The resulting 3-D cuboid information is described using the minimum and maximum values of row, column, and slice axes. Figure 3 illustrates a flow diagram for the 2-D bounding boxes to the 3-D cuboid conversion process.

### 3-D false positives reduction

Because generated 3-D breast nodule cuboids may include redundant outcomes and FPs, we perform an additional FP reduction process based on chest wall region segmentation. For this purpose, the U-Net model^15^ with a residual network with 18-layer (ResNet-18)^26^ pre-trained on the ImageNet dataset^51^ as the encoder is utilized. The model training was performed using the auxiliary ABUS in-house dataset, where a sensitivity, specificity, and accuracy of $86.77\pm 4.15\%$, $91.75\pm 2.12\%$, and $89.99\pm 2.48\%$, respectively were achieved from a single run of subject-wise eight-fold cross-validation tests. In order to apply the model for the in-house ABUS datasets A and B, re-training was performed using the entire auxiliary dataset to secure the highest number of training samples. Those 3-D breast nodule cuboids with center coordinates located in the segmented chest wall regions are regarded as FPs and excluded for further processing. Additionally, redundant 3-D cuboids are aggregated using a hierarchical clustering technique with setting IoU criterion in a volumetric manner as a similarity metric. The aggregation procedure is similar to those in 2-D FP reduction but in a 3-D sense. Consequently, the resulting 3-D breast nodule cuboids are defined as final 3-D breast nodules detected and used for performance assessment.

## Results

### Evaluation protocols

In our experiments, breast nodule detection performance is assessed for ABUS images in BI-RADS categories 3, 4, and 5. Firstly, we performed a single run of ten-fold cross-validation tests using the in-house ABUS dataset A. During the training phase, a single run of ten-fold hold-out validation was performed using the training set. The data partitioning tasks were performed based on the patient. Secondly, we performed a hold-out test using the best model determined from the cross-validation tests using the in-house ABUS dataset B. To keep as much as initial detection outcomes and remove undesirable outcomes at false positives reduction stages, we set thresholds $\tau _i$ and $\tau _s$ at 0.3 and 5 (corresponding to $1\,\textrm{mm}$) heuristically during the 2-D bounding boxes to 3-D cuboids conversion stage.

In our study, a detection outcome was considered a true positive (TP) if the center coordinate of the detected cuboid is within a ground truth (GT) cuboid according to Yap et al.^24^. There are other approaches utilizing the distance between the center coordinates of TP and GT cuboids (or bounding boxes for 2-D detection). For example, Chiang et al.^32^ and Moon et al.^31^ set $10\,\textrm{mm}$ and $15\,\textrm{mm}$ as the criteria. Hence, we also present the average and standard deviation of the distance between the center coordinates of TP and GT cuboids. For performance metrics, we employed the sensitivity ($\%$), the number of TP nodules ($\#$TPs), the number of GT nodules ($\#$GTs), precision ($\%$), the number of false positives (FPs) per pass, and the number of detected cuboids ($\#$DETs) per pass. Performance evaluation using these metrics was performed for (1) the entire data, (2) each BI-RADS category (BI-RADS categories 3, 4, and 5, separately), (3) a group of BI-RADS categories (suspicious nodules in BI-RADS categories 4 and 5), and (4) data without targetted breast nodules for detection (controlled group). A single run of cross-validation tests with a single run of hold-out validation using only the training set was performed for the in-house ABUS dataset A. The in-house ABUS dataset B was utilized as a separate test dataset to deal with the lack of data for external validation, where a single run of hold-out test was performed for the in-house ABUS dataset B. For the results from the cross-validation tests, a paired t-test^52,53^ was performed to demonstrate whether the improvement via 3-D FPs reduction is statistically significant (significance level = 0.01), and the differences among three plane images are statistically significant (significance level = 0.05). The statistical significance test was not performed for the hold-out test using the in-house ABUS dataset B because we obtained a single set of results from the test.

### Confidence threshold value selection

**Figure 4:**
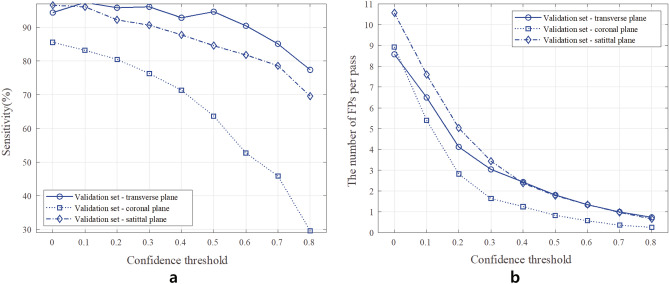
The sensitivity and number of FPs per pass with respect to the confidence threshold from experiments using the in-house ABUS datasets A and B. (**a**) sensitivity ($\%$). (**b**) the number of FPs per pass.

To decide the confidence threshold value, we set a range between 0 and 0.8 with an increment of 0.1 and evaluated the detection performance. Figure 4 shows average values of the sensitivity and the number of FPs per pass measured at the validation phase during the cross-validation tests. Considering the trade-off between the sensitivity and the number of FPs, we selected the confidence threshold value $\tau _c$, when the number of FPs is less than 2. As a result, the confidence threshold $\tau _c$ was chosen at 0.5 for transverse and sagittal plane images. Because the coronal plane images showed much lower sensitivity than those from other plane images at $\tau _c=0.5$, the confidence threshold for the coronal plane images was selected using the sensitivity values of the transverse and sagittal plane images at $\tau _{c}=0.5$. Consequently, a sensitivity of around $85\%$, which corresponds to the minimum value among those of the other plane images, was utilized to choose $\tau _c$ for the coronal plane images. These result in obtaining $\tau _{c}=0.5$ for the transverse and sagittal plane images and $\tau _c=0$ for the coronal plane images, respectively. Hereafter, the breast performance evaluation and analysis regarding performance enhancement via chest wall region segmentation were based on the selected operating point of the confidence values.

### Comprehensive performance evaluation

The sensitivity values and the number of TP and GT nodules measured from the cross-validation tests using the in-house ABUS datasets A detecting breast nodules in BI-RADS 3, 4, and 5 are presented in Table 1. The same sensitivity values were obtained before and after applying the 3-D FPs reduction for the entire experiment. Table 2 shows the number of FPs per pass and the precision values measured using the in-house datasets A. Due to the trade-off between the sensitivity and the precision, low precision values were obtained at the selected operating point. Hence, we utilize the number of FPs per pass directly to discuss how precisely our model detects breast nodules, and also present the number of detected cuboids. For all cases, the performance improvement of the number of FPs per pass by 3-D FPs reduction was statistically significant (significance level = 0.01). From the cross-validation tests using the in-house ABUS dataset A, the transverse plane images showed an average sensitivity of $90.98\%$ at 11.6 average FPs per pass after applying 3-D FPs reduction. There exist 15.2 ground-truth nodules and 12.2, 15.4, and 9.6 detected cuboids per plane in the test sets on average. The coronal ($84.41\%$ sensitivity at 14.7 FPs per pass) and sagittal ($83.38\%$ at 8.9 FPs per pass) plane images showed lower sensitivity than the transverse plane images. Among the three plane images, the transverse plane images showed a significantly better sensitivity performance than the sagittal plane images (significance level = 0.05). The other sensitivity comparisons between planes were not statistically significant. The sagittal plane images showed a significantly smaller number of FPs per pass than the coronal plane images (significance level = 0.05), while there was no statistically significant difference for the remaining comparisons between planes.

**Table 1:** Sensitivity ($\%$), the number of true positive nodules ($\#$TPs), the number of ground-truth nodules ($\#$GTs) measured using the in-house ABUS datasets A and B.

Nodule category	ABUS dataset	Sensitivity ($\%$)			$\#$TPs / $\#$GTs		
		Transverse	Coronal	Sagittal	Transverse	Coronal	Sagittal
Total	A	$90.98\pm 7.29$	$84.41\pm 13.20$	$83.38\pm 10.38$	13.9 / 15.2	12.8 / 15.2	12.7 / 15.2
	B	93.65	87.30	87.30	59 / 63	55 / 63	55 / 63
BI-RADS 3	A	$85.24\pm 21.44$	$80.79\pm 19.16$	$79.64\pm 16.91$	4.1 / 4.7	3.8 / 4.7	3.8 / 4.7
	B	91.30	78.26	69.57	21 / 23	18 / 23	16 / 23
BI-RADS 4	A	$88.73\pm 20.46$	$85.09\pm 15.98$	$82.72\pm 14.85$	7.6 / 8.3	6.8 / 8.3	6.8 / 8.3
	B	94.12	91.18	97.06	32 / 34	31 / 34	33 / 34
BI-RADS 5	A	$100.00\pm 0.00$	$100.00\pm 0.00$	$94.44\pm 16.67$	2.2 / 2.2	2.2 / 2.2	2.1 / 2.2
	B	100.00	100.00	100.00	6 / 6	6 / 6	6 / 6
Suspicious	A	$90.91\pm 15.41$	$87.55\pm 14.19$	$85.13\pm 11.02$	9.8 / 10.5	9.0 / 10.5	8.9 / 10.5
	B	95.00	92.50	97.50	38 / 40	37 / 40	39 / 40

Performance values are reported for a set of nodule groups, such as total nodules (BI-RADS categories 3, 4, and 5), each BI-RADS category, and suspicious nodules (BI-RADS categories 4 and 5). Mean (sensitivity, $\#$TPs, and $\#$GTs) and standard deviation (sensitivity) values are reported for the in-house ABUS dataset A, and a single value is reported for the in-house ABUS dataset B.

**Table 2:** The number of false positives ($\#$FPs) per pass, the number of detected cuboids ($\#$DETs) per pass, and the precision ($\%$) measured using the in-house ABUS datasets A and B for detection of total nodules (BI-RADS categories 3, 4, and 5).

Nodule category	3-D FPs reduction	ABUS dataset	$\#$FPs per pass ($\#$DETs per pass)			Precision ($\%$)		
			Transverse	Coronal	Sagittal	Transverse	Coronal	Sagittal
Total	No	A	$14.1\pm 5.6$ ($14.7\pm 5.7$)	$24.1\pm 12.2$ ($24.7\pm 12.2$)	$11.9\pm 3.7$ ($12.6\pm 3.8$)	$4.69\pm 2.56$	$3.30\pm 2.08$	$5.56\pm 2.96$
		B	10.8 (11.2)	13.9 (14.4)	16.6 (17.0)	4.13	3.77	3.25
	Yes	A	$11.6\pm 5.6^{*}$ ($12.2\pm 5.7$)	$14.7\pm 6.3^{*}$ ($15.4\pm 6.3$)	$8.9\pm 4.3^{*}$ ($9.6\pm 4.5$)	$5.81\pm 3.09$	$4.88\pm 2.63$	$7.49\pm 3.47$
		B	8.6 (9.0)	9.9 (10.4)	16.0 (16.4)	5.18	5.16	3.38

Mean and standard deviation values are reported for the in-house ABUS dataset A, and a single value is reported for the in-house ABUS dataset B. The symbol $^{*}$ denotes that the performance improvement via 3-D FPs reduction is statistically significant ($p<0.001$).

The hold-out test results using the in-house ABUS dataset B for detecting breast nodules in BI-RADS categories 3, 4, and 5 are presented in Tables 1 and 2. The transverse plane images showed the best results ($93.65\%$ sensitivity at 8.6 FPs per pass). The coronal plane images ($87.30\%$ sensitivity at 9.9 FPs per pass) showed smaller FPs per pass than the sagittal plane images ($87.30\%$ sensitivity at 16.0 FPs per pass). There are 63 GT nodules in total, and 9.0, 10.4, and 16.4 detected cuboids for the transverse, coronal, and sagittal planes. The 3-D FPs reduction produced decrease of around 2.2, 4.0, and 0.6 FPs per pass for the three planes. In addition to the performance assessment, the average distance between the center coordinates of TP and GT cuboids for the in-house ABUS datasets A and B is presented in Table 3. The average distance ranges from $3.50\,\textrm{mm}$ to $4.55\,\textrm{mm}$ for the in-house ABUS dataset A and $3.25\,\textrm{mm}$ to $4.37\,\textrm{mm}$ for the in-house ABUS dataset B. There values are less than the criteria reported in the literature (e.g., $10\,\textrm{mm}$^32^ and 15 mm^31^). Comprehensively less than $4.24\%$ of the entire slices in each volume contain TP or FP detection outcomes for the three views on average (in-house ABUS dataset A: 2.39–$2.99\%$, and in-house ABUS dataset B: 1.42–$4.24\%$). Hence, given the sensitivity performance shown in Table 1, the radiologists need to read less than $5\%$ of the entire images in each volume for interpretation. Applying the proposed CADe system may result in assistance via obtaining candidate nodule regions in an automatic manner and conserving reading time drastically.

**Table 3:** The distance between the center coordinates of true positive (TP) and ground truth (GT) cuboids in millimeters for BI-RADS categories 3, 4, and 5 nodule detection.

ABUS Dataset	Distance between Centers of TP and GT Cuboids ($\textrm{mm}$)		
	Transverse	Coronal	Sagittal
A	$3.50\pm 2.45$	$4.03\pm 3.15$	$4.55\pm 3.21$
B	$3.25\pm 2.40$	$4.20\pm 3.72$	$4.37\pm 3.07$

Mean and standard deviation values are reported.

### Performance evaluation per BI-RADS category

The sensitivity and the number of TP and GT nodules per each BI-RADS category measured using the in-house datasets A are presented in Table 1. Table 4 shows the number of FPs and DETs per pass and the precision measured from the cross-validation tests using the in-house ABUS dataset A per each BI-RADS category. As the results for the total BI-RADS categories, no sensitivity performance degradation was observed for the 3-D FPs reduction, and the performance improvement by the 3-D FPs reduction for all cases was statistically significant (significance level = 0.01). As the BI-RADS category increases, the sensitivity performance increases for all planes. For the cross-validation tests using the in-house ABUS datasets A, we obtained average sensitivities of $85.24\%$ at 13.3 FPs per pass (transverse, 14.6 detected cuboids), $80.79\%$ at 14.1 FPs per pass (coronal, 15.3 detected cuboids), and $79.64\%$ at 9.3 FPs per pass (sagittal, 10.6 detected cuboids) for volumes with BI-RADS category 3. For BI-RADS category 4, sensitivities of $88.73\%$ at 12.0 FPs per pass (transverse, 13.4 detected cuboids), $85.09\%$ at 12.4 FPs per pass (coronal, 14.1 detected cuboids), and $82.72\%$ at 6.9 FPs per pass (sagittal, 8.4 detected cuboids) were observed on average. For volumes with nodules in BI-RADS category 5, all GT nodules were successfully detected for the transverse and coronal planes with 13.2 and 19.3 FPs per pass among 15.0 and 21.5 detected cuboids, respectively. At the same time, a sensitivity of $94.44\%$ at 9.8 FPs per pass was achieved for the sagittal plane images (12.1 detected cuboids). For each category, the sagittal plane images outperformed the coronal plane images in terms of the number of FPs per pass with statistical significance (significance level = 0.05). There was no other statistically significant difference between planes.

From the hold-out test using the in-house ABUS dataset B, sensitivities of $91.30\%$ at 15.6 FPs per pass, $78.26\%$ at 18.1 FPs per pass, and $69.57\%$ at 23.9 FPs per pass were obtained for the images of the transverse, coronal, and sagittal planes in BI-RADS category 3 (Tables 1 and 4). There were 16.8, 19.1, and 25.1 detected cuboids for the transverse, coronal, and sagittal planes, and 23 GT nodules for BI-RADS category 3. For scans with nodules in BI-RADS category 4, we achieved sensitivities of $94.12\%$ at 10.4 FPs per pass (transverse, 11.9 detected cuboids), $91.18\%$ at 13.3 FPs per pass (coronal, 15.1 detected cuboids), and $97.06\%$ at 19.7 FPs per pass (sagittal, 21.6 detected cuboids). All GT nodules within BI-RADS category 5 (6 GT nodules) were successfully detected with 3.8, 7.3, and 11.0 FPs per pass among 4.8, 9.3, and 12.2 detected cuboids for the transverse, coronal, and sagittal planes. As in the cross-validation tests using the in-house ABUS dataset A, the sensitivity value rises as the BI-RADS category increases.

**Table 4:** The number of false positives ($\#$FPs) per pass, the number of detected cuboids ($\#$DETs) per pass, and the precision ($\%$) measured using the in-house ABUS datasets A and B per volumes in each BI-RADS category.

Nodule Category	3-D FPs Reduction	ABUS Dataset	$\#$FPs per Pass ($\#$DETs per Pass)			Precision ($\%$)		
			Transverse	Coronal	Sagittal	Transverse	Coronal	Sagittal
BI-RADS 3	No	A	$15.4\pm 7.4$ ($16.7\pm 7.1$)	$23.9\pm 13.3$ ($25.1\pm 13.5$)	$12.4\pm 4.9$ ($13.7\pm 5.2$)	$10.64\pm 10.25$	$6.01\pm 4.23$	$9.91\pm 4.83$
		B	11.1 (12.2)	16.6 (17.5)	19.2 (20.1)	9.13	5.37	4.59
	Yes	A	$13.3\pm 7.0^{*}$ ($14.6\pm 6.8$)	$14.1\pm 8.5^{*}$ ($15.3\pm 8.6$)	$9.3\pm 5.2^{*}$ ($10.6\pm 5.4$)	$12.35\pm 11.20$	$17.68\pm 29.54$	$13.52\pm 6.51$
		B	15.6 (16.8)	18.1 (19.1)	23.9 (25.1)	7.30	5.46	4.88
BI-RADS 4	No	A	$13.7\pm 10.1$ ($15.1\pm 10.7$)	$21.4\pm 10.1$ ($23.2\pm 9.8$)	$9.2\pm 4.4$ ($10.8\pm 4.7$)	$16.15\pm 21.18$	$8.96\pm 5.30$	$16.40\pm 8.85$
		B	12.0 (13.5)	16.7 (18.5)	20.2 (22.2)	11.08	9.91	8.86
	Yes	A	$12.0\pm 10.4^{*}$ ($13.4\pm 11.0$)	$12.4\pm 5.7^{*}$ ($14.1\pm 5.5$)	$6.9\pm 4.1^{*}$ ($8.4\pm 4.5$)	$20.37\pm 28.57$	$14.30\pm 8.13$	$20.56\pm 8.10$
		B	10.4 (11.9)	13.3 (15.1)	19.7 (21.6)	12.61	12.14	9.09
BI-RADS 5	No	A	$14.1\pm 10.5$ ($15.8\pm 10.5$)	$27.1\pm 17.3$ ($29.3\pm 18.1$)	$11.0\pm 9.4$ ($13.4\pm 10.1$)	$18.63\pm 18.67$	$8.57\pm 4.52$	$22.44\pm 13.90$
		B	4.5 (5.5)	11.0 (13.0)	11.5 (12.7)	18.18	15.38	9.21
	Yes	A	$13.2\pm 9.7^{*}$ ($15.0\pm 9.7$)	$19.3\pm 13.2^{*}$ ($21.5\pm 13.9$)	$9.8\pm 8.3^{*}$ ($12.1\pm 9.0$)	$19.11\pm 18.53$	$12.42\pm 6.82$	$24.32\pm 14.70$
		B	3.8 (4.8)	7.3 (9.3)	11.0 (12.2)	20.69	21.43	9.59

Mean and standard deviation values are reported for the in-house ABUS dataset A, and a single value is reported for the in-house ABUS dataset B. The symbol $^{*}$ denotes that the performance improvement via 3-D FPs reduction is statistically significant ($p<0.001$).

### Performance evaluation for volumes with suspicious nodules

The sensitivity and the number of TP and GT nodules measured from the cross-validation tests using the in-house ABUS dataset A for detecting suspicious nodules (BI-RADS categories 4 and 5) are reported in Table 1. Table 5 presents the number of FPs per pass, the number of detected cuboids per pass, and the precision measured from the cross-validation tests. The 3-D FPs reduction produced a statistically significant reduction of FPs (significance level = 0.01) without degrading the sensitivity for all cases. Sensitivities of $90.91\%$ at 12.1 FPs per pass (transverse, 13.6 detected cuboids), $87.55\%$ at 13.8 FPs per pass (coronal, 15.7 detected cuboids), and $85.13\%$ at 7.1 FPs per pass (sagittal, 8.9 detected cuboids) were observed, while there was no statistically significant difference between the sensitivities between planes. In terms of FPs per pass, the sagittal plane images significantly outperformed the coronal plane images (significance level = 0.05). There was no other statistically significant difference in FPs per pass between planes.

**Table 5:** The number of false positives ($\#$FPs) per pass, the number of detected cuboids ($\#$DETs) per pass, and the precision ($\%$) measured using the in-house ABUS datasets A and B for volumes with suspicious nodules (BI-RADS categories 4 and 5)

Nodule Category	3-D FPs Reduction	ABUS Dataset	$\#$FPs per Pass ($\#$DETs per Pass)			Precision ($\%$)		
			Transverse	Coronal	Sagittal	Transverse	Coronal	Sagittal
Suspicious	No	A	$13.6\pm 10.0$ ($15.1\pm 10.4$)	$22.5\pm 11.3$ ($24.4\pm 11.2$)	$9.1\pm 4.5$ ($10.8\pm 4.9$)	$16.79\pm 19.75$	$9.01\pm 4.94$	$18.48\pm 8.86$
		B	10.8 (12.2)	15.7 (17.6)	18.8 (20.6)	11.62	10.58	9.89
	Yes	A	$12.1\pm 10.0^{*}$ ($13.6\pm 10.4$)	$13.8\pm 6.9^{*}$ ($15.7\pm 6.8$)	$7.1\pm 4.1^{*}$ ($8.9\pm 4.5$)	$19.56\pm 23.17$	$13.99\pm 7.62$	$22.39\pm 8.34$
		B	9.3 (10.7)	12.3 (14.1)	18.2 (20.1)	13.21	13.16	9.14

Mean and standard deviation values are reported for the in-house ABUS dataset A, and a single value is reported for the in-house ABUS dataset B. The symbol $^{*}$ denotes that the performance improvement via 3-D FPs reduction is statistically significant ($p<0.001$).

The sensitivity, the number of FP and GT nodules measured from the hold-out test using the in-house ABUS dataset B for the suspicious nodules are presented in Table 1. The number of FPs per pass, the number of detected cuboids per pass, and the precision corresponding to the sensitivity value are provided in Table 5. As in the cross-validation tests, the 3-D FPs reduction retained the original sensitivity performance. For 40 GT nodules with suspicious nodules (BI-RADS categories 4 and 5), sensitivities of $95.00\%$ at 9.3 FPs per pass (transverse, 10.7 detected cuboids), $92.50\%$ at 12.3 FPs per pass (coronal, 14.1 detected cuboids), and $97.50\%$ at 18.2 FPs per pass (sagittal, 20.1 detected cuboids). Compared to the results for scans with benign-appearing nodules in BI-RADS category 3, detecting nodules with suspicious features is regarded to be relatively easy due to their distinct appearances.

### Performance evaluation for controlled group

Our study aims to detect breast nodules within BI-RADS categories 3, 4, and 5 due to their clinical impact. Because the in-house ABUS datasets A and B contain nodules in BI-RADS category 2, and the in-house ABUS dataset B includes scans without nodules, subsets of them are utilized as a controlled group. In these subsets, all detection outcomes are considered FPs. Table 6 presents the number of FPs per pass measured using the subsets. The performance improvement via 3-D FPs reduction was statistically significant (significance level = 0.01) for all plane images of the in-house ABUS dataset A. For 17 scans in the in-house ABUS dataset A with breast nodules in BI-RADS category 2, 5.8, 9.6, and 6.6 FPs per pass were obtained for the transverse, coronal, and sagittal planes by 3-D FPs reduction. We achieved 8.1 (transverse), 9.8 (coronal), and 15.5 (sagittal) FPs per pass for 36 scans in the in-house ABUS dataset B with breast nodules in BI-RADS category 2. For 80 scans without nodules in the in-house ABUS dataset B, 7.3, 7.5, and 13.6 FPs per pass were observed for the transverse, coronal, and sagittal planes. These values are slightly lower than those measured for total scans, as reported in Table 2.

**Table 6:** The number of false positives ($\#$FPs) per pass measured using partial volumes of the in-house ABUS datasets A and B.

3-D FPs reduction	ABUS dataset	The number of false positives (FPs) per pass		
		Transverse	Coronal	Sagittal
No	A1	$9.0\pm 5.1$	$15.4\pm 3.1$	$11.8\pm 7.6$
	B1	9.6	13.1	16.0
	B2	10.5	12.1	14.4
Yes	A1	$5.8\pm 3.6^{*}$	$9.6\pm 3.3^{*}$	$6.6\pm 4.4^{*}$
	B1	8.1	9.8	15.5
	B2	7.3	7.5	13.6

A1 (17 scans) and B1 (36 scans) are subsets of the in-house ABUS datasets A and B, respectively, where the scans contain nodules solely in BI-RADS category 2. B2 (80 scans) denotes a subset of the in-house ABUS dataset B, where the scans have no nodule. The symbol $^{*}$ denotes that the performance improvement via 3-D FPs reduction is statistically significant ($p<0.001$).

### Detection outcomes visualization

**Figure 5:**
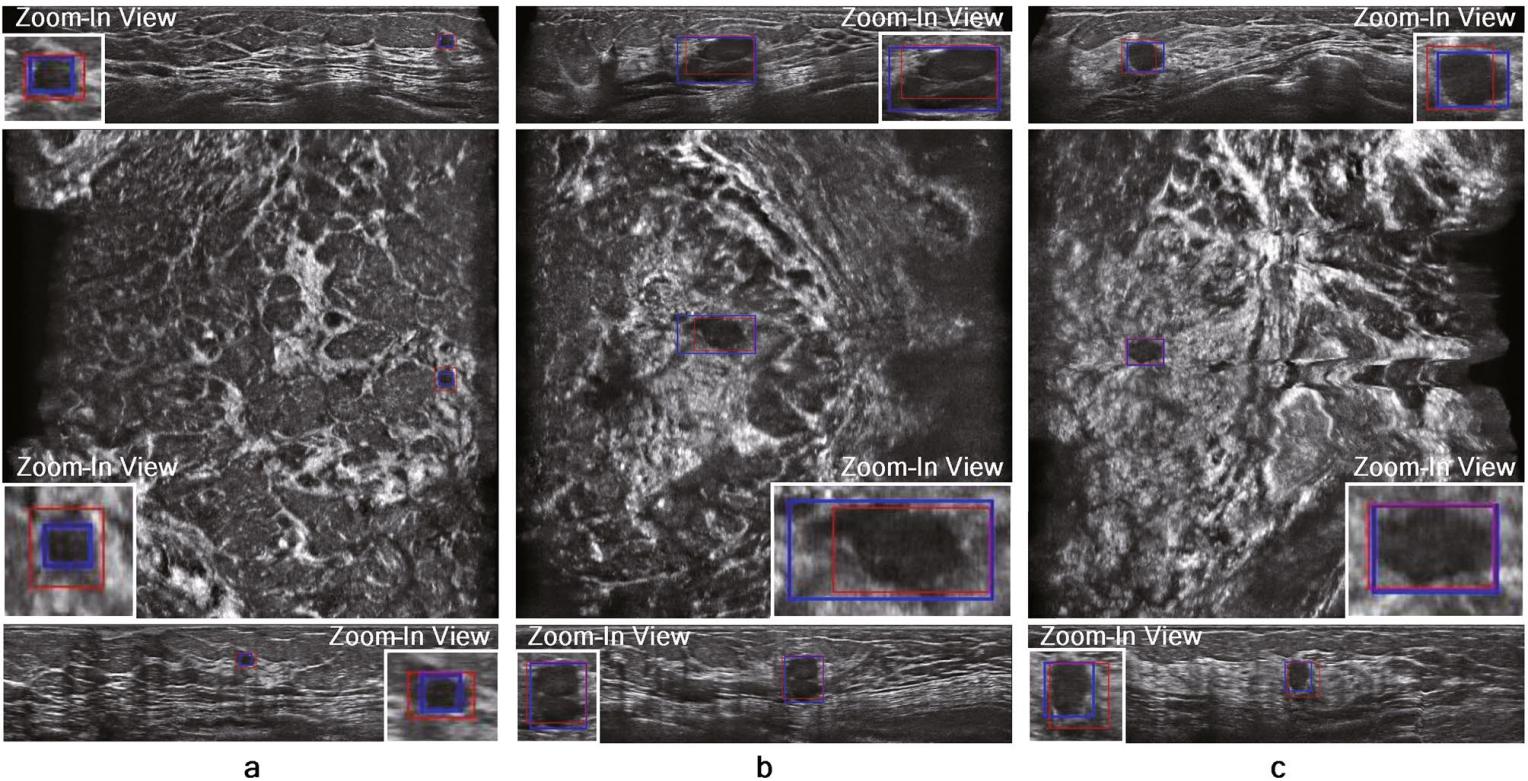
TP results on the in-house ABUS dataset A. (**a**) BI-RADS category 3 case. (**b**) BI-RADS category 4 case. (**c**) BI-RADS category 5 case. Images are arranged in the order of transverse, coronal, and sagittal views in a downward direction. Blue boxes with a thick solid line show the ground truth bounding box, and red boxes with a thin solid line show the TP detection.

**Figure 6:**
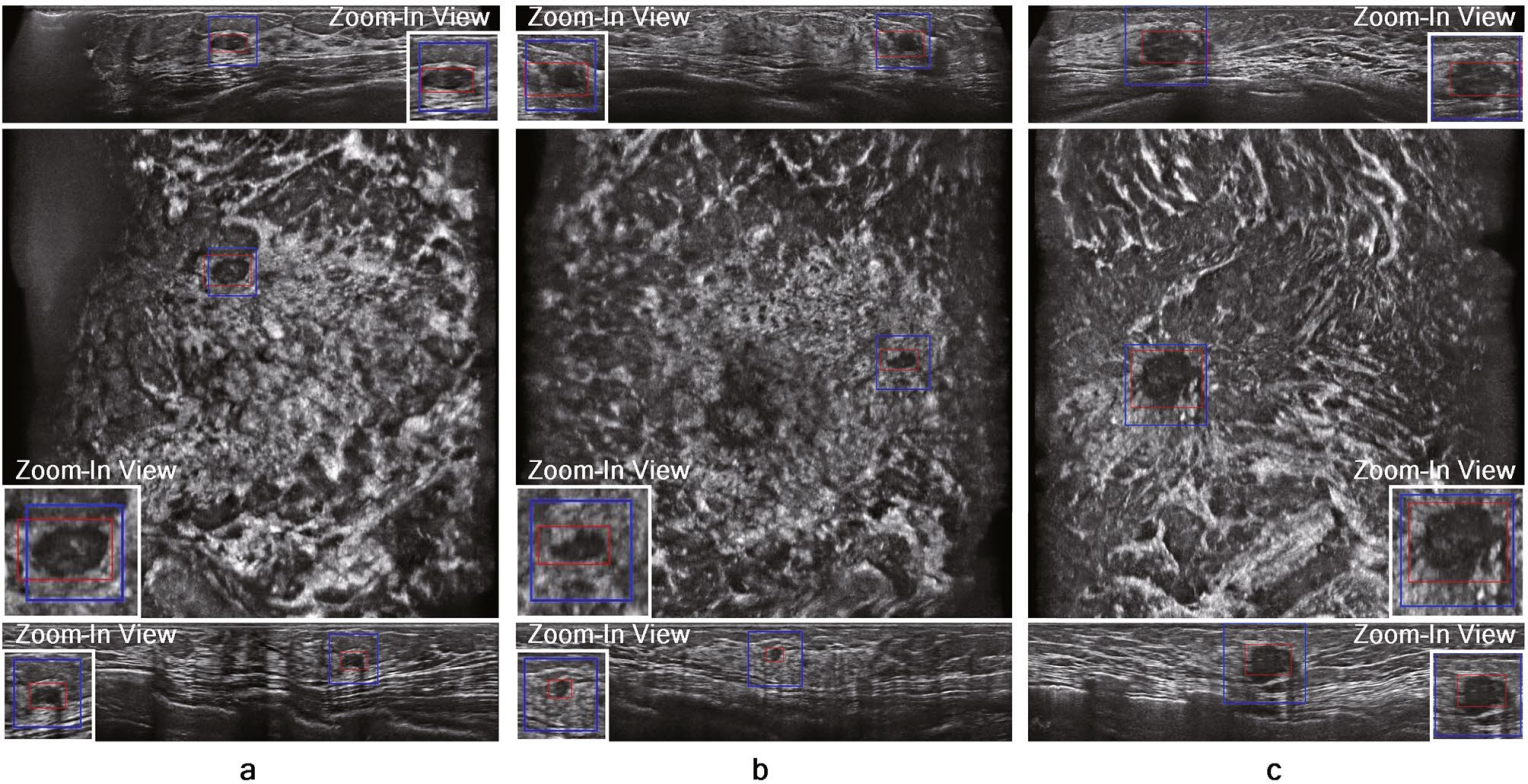
TP results on the in-house ABUS dataset B. (**a**) BI-RADS category 3 case. (**b**) BI-RADS category 4 case. (**c**) BI-RADS category 5 case. Images are arranged in the order of transverse, coronal, and sagittal views in a downward direction. Blue boxes with a thick solid line show the ground truth bounding box, and red boxes with a thin solid line show the TP detection.

**Figure 7:**
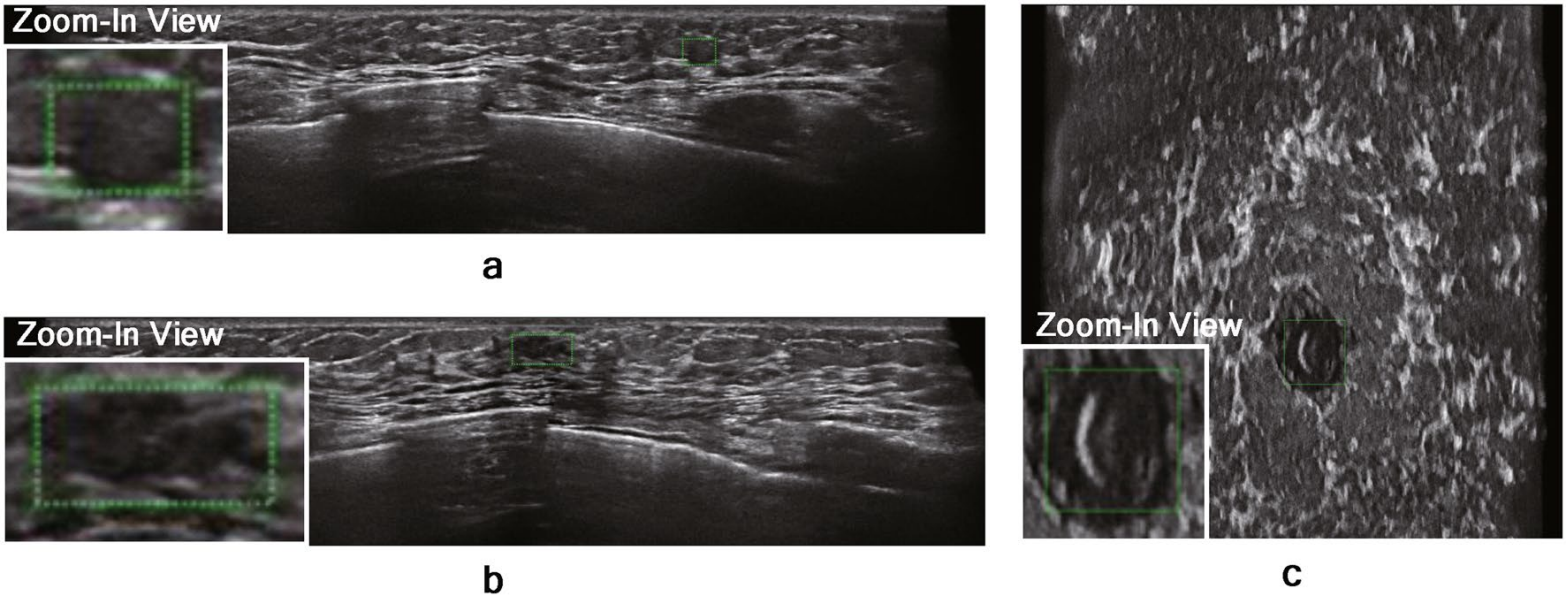
FP results on the in-house ABUS dataset A and B. (**a**) fat lobule (transverse view). (**b**) acoustic shadowing (transverse view). (**c**) nipple (coronal view). Green boxes with a dotted line show the FP detection.

The TP results on the in-house ABUS dataset A and B are illustrated in Figs. 5 and 6, respectively. The nodules with relatively large sizes are easily distinguishable from the background tissue (see BI-RADS categories 4 and 5 cases in Figs. 5 and 6). Even the relatively small nodules in BI-RADS category 3 show clear separation from the background tissue for TP results. Figure 7 illustrates the FP results on the in-house ABUS datasets. The FP results include the detection of fat lobules, acoustic shadowing, and nipple, where the fat lobules and acoustic shadowing are the majority of FPs.

## Discussion

In this study, we proposed a breast nodule detection system on ABUS volumes based on the Faster R-CNN and U-Net models. The breast nodules were detected in the form of a 3-D cuboid utilizing each of the transverse, coronal, and sagittal plane images separately. The hierarchical clustering is utilized to reduce 2-D FPs. Subsequently, 3-D cuboids are generated from sequential 2-D bounding boxes based on a set of operations. Consequently, the 3-D FPs in the chest wall regions are removed from the detection outcomes. From the cross-validation tests using the in-house ABUS dataset A containing breast nodules with BI-RADS categories 3, 4, and 5, we achieved average sensitivities of $90.98\%$, $84.41\%$, and $83.38\%$ for transverse, coronal, and sagittal plane images, respectively. At such sensitivity values, 11.6, 14.7, and 8.9 FPs per pass were achieved on average among 12.2, 15.4, and 9.6 detected cuboids. For the cross-validation tests, the improvement of the number of FPs per pass via 3-D FPs reduction was statistically significant without deteriorating the sensitivity. Additionally, the transverse plane images outperformed the sagittal plane images with statistical significance in terms of sensitivity. In view of the number of FPs per pass, the sagittal plane images showed better performance than the coronal plane images with statistical significance. The same statistical significance test results were obtained for data in each BI-RADS category and with suspicious nodules. For the hold-out test using the in-house ABUS dataset B, we achieved sensitivities of $94.65\%$, $87.30\%$, and $87.30\%$ for the transverse, coronal, and sagittal plane images. Concurrently, 8.6, 9.9, and 16.0 FPs per pass were obtained among 9.0, 10.4, and 16.4 detected cuboids, respectively, at such sensitivity values. The average distances between TP and GT cuboids were within the ranges $\left[ 3.50,4.55\right]$ and $\left[ 3.25,4.37\right]$ millimeters for the in-house ABUS datasets A and B, where the values are smaller than the criteria reported in the literature^31,32^. For both datasets, detecting breast nodules with a higher BI-RADS category was relatively easy due to their distinct appearance. Finally, the performances result in less than $5\%$ of the entire slices in a volume containing detection outcomes regardless of TPs or FPs. Hence, the proposed 3-D breast nodule detection system can drastically assist in saving time and effort for reading by radiologists.

Comparisons with the state-of-the-art 3-D breast nodule detection methods in the literature in terms of the sensitivity and the number of FPs per pass are presented in Table 7. Methods based on 3-D CNN (Chiang et al.^32^ and Moon et al.^31^) show a certain level of good sensitivity performance (over $95.00\%$), while a relatively high FPs per pass (21.6) was reported by Moon et al.^31^. Compared with the study by Chiang et al.^32^ (2.3 FPs per pass), our method for data with BI-RADS categories 3, 4, and 5 shows lower sensitivity and higher FPs per pass. However, they included a much larger number of malignant tumors than benign tumors, where the detection complexity is considered relatively low. Similarly, we obtained lower sensitivity and the number of FPs per pass than the study by Zhou et al.^38^ based on a modified Faster R-CNN model, where the average tumor volume ($2\,\text{cm}^{3}$) is considered high, and the number of patients is relatively small. Compared with the convolutional BiLSTM proposed by Malekmohammadi et al.^37^, we obtained lower sensitivity with smaller FPs per pass for total data. However, they utilized a small dataset containing a much larger number of malignant nodules than benign nodules. Zhang et al.^35^ reported internal and external validation results by applying the YOLOv5 model on the biggest dataset consisting of a much larger number of benign nodules than malignant nodules. Overall, our method obtained better sensitivity performances than the study by Zhang et al.^35^, but with higher FPs per pass. In view of the dataset composition, our study is considered to handle a 3-D breast nodule detection problem with relatively higher complexity than other studies except for the study by Zhang et al.^29^.

**Table 7:** Performance comparisons with state-of-the-art methodologies in the literature in terms of the sensitivity (SEN, $\%$) and the number of false positives ($\#$FPs per pass).

Method	$\#$Patients	Ground truth	Validation	Nodule	SEN ($\%$)	$\#$FPs per Pass	Note
Chiang et al.^32^	187	Pathology-proven	Internal	Benign+Malignant	80.00	0.6	60 benign and 240 malignant tumors
					95.00	2.3	
Moon et al.^31^	246	Pathology-proven	Internal	Benign+Malignant	84.80	3.7	79 benign and 254 malignant tumors
					98.10	21.6	
Zhang et al.^35^	741	Pathology-proven	Internal	BI-RADS 2,3,4,5	78.10	–	2896 benign and 218 malignant lesions
				BI-RADS 2	74.70	4.0	
				BI-RADS 3	79.70		
				BI-RADS 4,5	96.50		
			External	BI-RADS 2,3,4,5	71.20	–	
				BI-RADS 2	88.40	4.0	
				BI-RADS 3	74.70		
				BI-RADS 4,5	95.80		
Malekmohammadi et al.^37^	43	Pathology-proven	Internal	Benign+Malignant	60.00	0.3	13 benign and 42 malignant mass
					100.00	16.0	
Zhou et al.^38^	75	Pathology-proven	Internal	Benign+Malignant	90.12	5.4	Average tumor volume $=2cm^{3}$
					95.06	0.6	
Proposed	131	BI-RADS categorization	Internal	BI-RADS 3,4,5	90.98	11.6	78, 124, and 25 nodules in BI-RADS categories 3, 4, and 5
				BI-RADS 3	85.24	13.3	
				BI-RADS 4	88.73	12.0	
				BI-RADS 5	100.00	13.2	
				BI-RADS 4,5	90.91	12.1	
			External (alternative)	BI-RADS 3,4,5	93.65	10.8	
				BI-RADS 3	91.30	15.6	
				BI-RADS 4	94.12	10.4	
				BI-RADS 5	100.00	3.8	
				BI-RADS 4,5	95.00	9.3	

One of the limitations of our study is that our system tends to generate a substantial number of FPs even within the controlled group (5.8–15.5 FPs) because the system is tailored towards generating high amounts of detections. We note that this tendency may not pose a significant issue at the system deployment stage since the system is intended to be utilized by radiologists to reduce the interpretation time primarily, and potential adjustments of the operating threshold can be made for specific application scenarios. Another limitation of our system is that there is no process to reduce FPs regarding fat lobule and acoustic shadowing. We believe filtering out most of these regions does not engage a complex methodology. Additionally, we substitute external validation using experiments on a dataset acquired from the same institute but in different acquisition periods due to a lack of data availability. Moreover, the auxiliary ABUS dataset is regarded as relatively small (eight subjects and 480 images), where retaining more data for segmenting the chest wall regions is selected as one of our immediate future works. Our future works include handling the FPs, such as fat lobule and acoustic shadowing, modifying the Faster R-CNN model to be more suitable for breast nodule detection, and performing external validation.

In this study, we proposed a 3-D breast nodule detection system using a simple 2-D Faster R-CNN model. A threshold operation on confidence values and aggregation of redundant 2-D bounding boxes were performed to reduce 2-D FPs. Subsequently, to build 3-D breast nodule information using 2-D detection outcomes, we presented 2-D bounding boxes to a 3-D cuboid conversion module. Additionally, 3-D cuboids in the chest wall region were removed based on a U-Net model. Similar to the 2-D case, redundant 3-D cuboids were aggregated to reduce 3-D FPs. Our experiments using the in-house datasets A and B show encouraging results in terms of the sensitivity and the number of FPs.

## Data Availability

The datasets generated during and/or analyzed during the current study are not publicly available due to privacy or ethical restrictions but are available from the first author or the corresponding author on reasonable request.

## Code availability

The code for preprocessing and learning contains intellectual property and cannot be released publicly. The preprocessing module and Faster R-CNN algorithm were implemented based on https://albumentations.ai/docs/examples/pytorch_classification/ and https://pytorch.org/vision/stable/_modules/torchvision/models/detection/faster_rcnn.html, respectively.
